# The MGED Ontology: A Framework for Describing Functional Genomics Experiments

**DOI:** 10.1002/cfg.234

**Published:** 2003-02

**Authors:** Christian J. Stoeckert, Helen Parkinson

**Affiliations:** 1 Department of Genetics and Center for Bioinformatics University of Pennsylvania 1415 Blockley Hall 423 Guardian Drive Philadelphia PA 19104 USA; 2 European Bioinformatics Institute EMBL Outstation Wellcome Trust Genome Campus Hinxton, Cambridge CB10 1SD UK

## Abstract

The Microarray Gene Expression Data (MGED) society was formed with an initial focus on experiments involving microarray technology. Despite the diversity of applications, there are common concepts used and a common need to capture experimental information in a standardized manner. In building the MGED ontology, it was recognized that it would be impractical to cover all the different types of experiments on all the different types of organisms by listing and defining all the types of organisms and their properties. Our solution was to create a framework for describing microarray experiments with an initial focus on the biological sample and its manipulation. For concepts that are common for many species, we could provide a manageable listing of controlled terms. For concepts that are species-specific or whose values cannot be readily listed, we created an ‘OntologyEntry’ concept that referenced an external resource. The MGED ontology is a work in progress that needs additional instances and particularly needs constraints to be added. The ontology currently covers the experimental sample and design, and we have begun capturing aspects of the microarrays themselves as well. The primary application of the ontology will be to develop forms for entering information into databases, and consequently allowing queries, taking advantage of the structure provided by the ontology. The application of an ontology of experimental conditions extends beyond microarray experiments and, as the scope of MGED includes other aspects of functional genomics, so too will the MGED ontology.


**Comparative and Functional Genomics**
*Comp Funct Genom* 2003; **4**: 127–132.
Published online in Wiley InterScience (www.interscience.wiley.com). **DOI:** 10.1002/cfg.234

**Conference Review**

### The MGED ontology: a framework for describing functional genomics experiments

Christian J. Stoeckert Jr[1]* and Helen Parkinson[2]

[1] *Department of Genetics and Center for Bioinformatics, University of Pennsylvania, 1415 Blockley Hall, 423 Guardian Drive, Philadelphia, PA 19104, USA*
[2] *European Bioinformatics Institute, EMBL Outstation, Wellcome Trust Genome Campus, Hinxton, Cambridge CB10 1SD, UK*

**Correspondence to:*
*Christian J. Stoeckert Jr, Department of Genetics and Center for Bioinformatics, University of Pennsylvania, 1415 Blockley Hall, 423 Guardian Drive, Philadelphia, PA 19104, USA.*
*E-mail: stoeckrt@pcbi.upenn.edu*

### Abstract

**The Microarray Gene Expression Data (MGED) society was formed with an initial focus on experiments involving microarray technology. Despite the diversity of applications, there are common concepts used and a common need to capture experimental information in a standardized manner. In building the MGED ontology, it was recognized that it would be impractical to cover all the different types of experiments on all the different types of organisms by listing and defining all the types of organisms and their properties. Our solution was to create a framework for describing microarray experiments with an initial focus on the biological sample and its manipulation. For concepts that are common for many species, we could provide a manageable listing of controlled terms. For concepts that are species-specific or whose values cannot be readily listed, we created an 'OntologyEntry' concept that referenced an external resource. The MGED ontology is a work in progress that needs additional instances and particularly needs constraints to be added. The ontology currently covers the experimental sample and design, and we have begun capturing aspects of the microarrays themselves as well. The primary application of the ontology will be to develop forms for entering information into databases, and consequently allowing queries, taking advantage of the structure provided by the ontology. The application of an ontology of experimental conditions extends beyond microarray experiments and, as the scope of MGED includes other aspects of functional genomics, so too will the MGED ontology. Copyright © 2003 John Wiley & Sons, Ltd.**

Received: 14 November 2002
Accepted: 19 November 2002

**Keywords:** **microarray; ontology; MGED**

### Introduction

Microarray technology is a highly parallel method to monitor the presence and/or abundance of biological molecules through hybridization to specific probes arrayed on a solid support, such as a glass slide. The original and most widely used application is for monitoring RNA abundance [17], which has been applied in a wide variety of organisms for a wide variety of purposes. In a typical microarray study, biological materials (biomaterials) are collected and perhaps treated in some fashion, RNA is extracted from these biomaterials, the RNA is copied into complementary DNA (cDNA) and labelled with fluorescent dyes or radioactivity for detection, the labelled cDNA is hybridized to an array, an image of the array is generated, and image intensities corresponding to the labelled cDNA are quantified. Much of the attention on microarray experiments has been focused on the analysis of these quantified intensities that represent gene expression. Proper interpretation of microarray experiments also requires an accurate, unambiguous description of all the steps leading to the generation of the quantified intensities. Descriptions of the biology and design of the experiment are as important as descriptions of the microarray design and usage. Many of these descriptions, such

Copyright © 2003 John Wiley & Sons, Ltd.

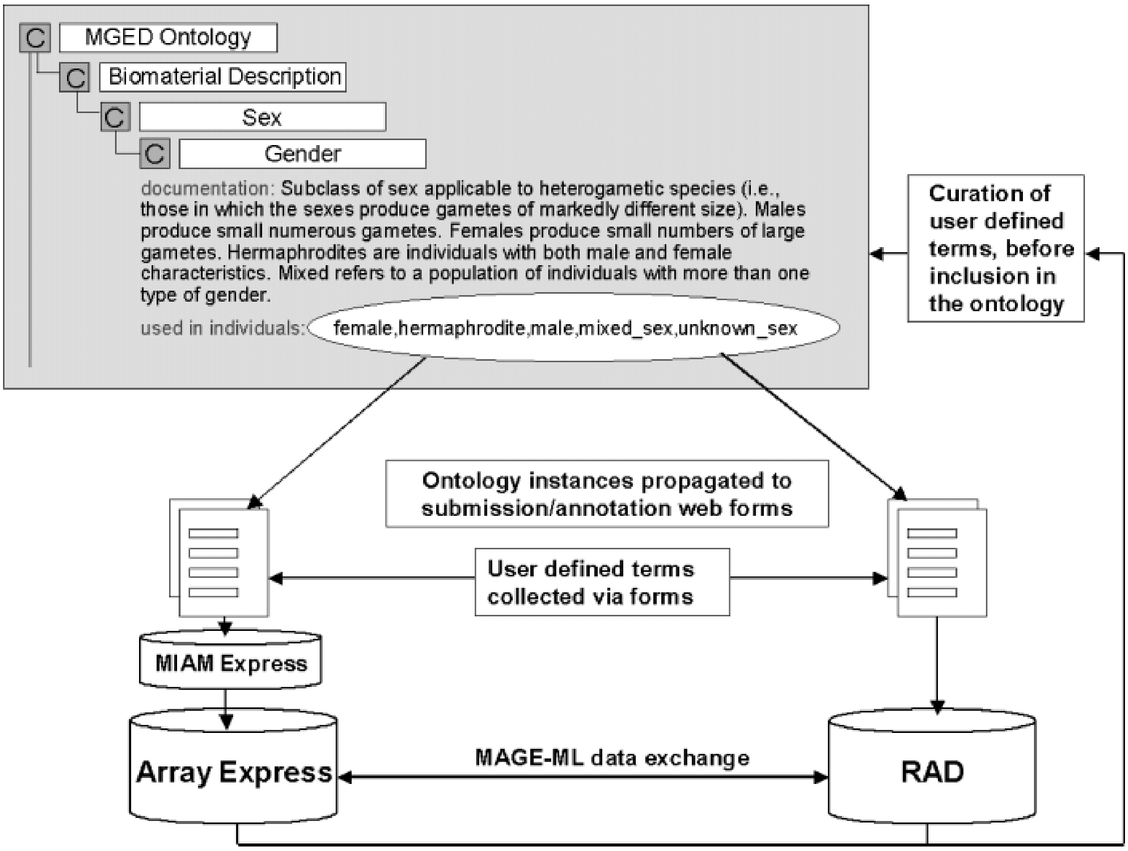

**Figure 1.** Instances from the MGED ontology (part of which is shown) are used in forms used by the ArrayExpress and RAD databases for data annotation and submission. New user defined instances gleaned from submissions feed back into the ontology after curation. Use of the ontology ensures that the databases are able to exchange meaningful data in MAGE-ML format as the details of protocols, are sufficiently captured in text. However, it is necessary to query on some descriptions (e.g. what organism was studied, what was its age, and what pathology was associated with it?) in order to find experiments of interest and group them appropriately. In order to query these types of descriptions, it is necessary to provide them in a form that computer programs can process and tell which are the same and which are different. Describing the relevant aspects of biological materials used in microarray experiments can be quite complex, and thus it is also important to be able to specify the relationships between these descriptions. For these reasons, an ontology for microarray experiments is needed.

Ontologies have come to mean different things to different people. We use the commonly cited usage of Gruber [7]. That is, the concepts used in our ontology are defined and the relationships between the concepts are specified. The ontology is being built as part of a coordinated effort by members of the microarray community through the Microarray Gene Expression Data (MGED) Society. MGED [12] has generated a set of guidelines for supplying the minimal information about a microarray experiment (MIAME, [3]). These guidelines have provided the foundation for concepts to be included in the ontology. A foundation for the relationships between the concepts was provided by the MAGE effort [19], developed jointly by MGED, Rosetta, and others. MAGE (microarray gene expression) is an object model that has been formally accepted as a standard by the Object Management Group (OMG, [15]) and implemented as a form of XML (MAGE-ML).

The purpose of the MGED ontology is to provide, either directly or indirectly, the terms

Copyright © 2003 John Wiley & Sons, Ltd. 

needed to follow the MIAME guidelines and referenced by MAGE. For example, MIAME asks that the organism part used is supplied (when relevant), and MAGE specifies how biomaterial characteristics such as organism part are encoded in a MAGE-ML document. The terms for organism part are not provided by either MIAME or MAGE, neither are terms for describing the age of the sample, the experimental design or the types of protocols used.

### An experimental ontology

The MGED ontology is an ontology of experiments, specifically microarray experiments, but potentially extensible to other types of functional genomics experiments. Although the major component of the ontology involves biological descriptions, it is not an ontology of molecular, cellular or organismal biology. Rather, it is an ontology that includes concepts of biological features relevant to the interpretation and analysis of an experiment. This distinction is critical for both establishing the scope of the ontology and providing direction in its construction.

Descriptions of biomaterials and their treatments become very complex, even when limited to aspects relevant to usage in a microarray experiment. For example, a study consisting of mice that have been given a compound in their drinking water to test the effects on their livers requires specifying what kind of mice, where they were obtained, how they are housed, and how the compound was delivered, in addition to the identity and dose of the compound. Details of the experimental design are needed as well, such as which data sets came from which mice, and whether livers were pooled or split. These are all needed to interpret the data obtained, as well as to reproduce the experiment.

Experimental descriptions can be thought of as falling into three categories: the types of information (classes) that need to be captured, their properties (attributes) and the actual values (instances) used. In our hypothetical study, we need to have categories or classes for 'organism' to indicate that mice were used, for 'compound' to indicate which substance (or chemical) was used, and for 'treatment' to indicate how the compound was administered to the mice. We also need classes for the age, sex, strain and other characteristics that may contribute to, or influence, the effects of the treatment. Furthermore, we need classes for the experimental, or study, design and what were the factors of interest (e.g. the compound). Finally, as this is a microarray experiment, we need classes for the different types of protocols, arrays, hardware and software that were used. As our goal is to facilitate queries, we do not need to capture everything about a microarray experiment, but we do need to provide a way to unambiguously and consistently describe relevant points, such as what kind of array platform was used and whether protocols of a particular type are provided.

The classes needed for a microarray experiment ontology can be further broken down into two categories. Some classes, such as those referring to parts of the microarray experiment and certain features common to many experiments, can be well described in terms of properties, values and subclasses (and their properties and values). For example, 'treatment' can be subclassed to distinguish compound-based treatments from treatments involving behavioural stimuli. Compound-based treatments can be given properties, such as delivery method, measurement of the compound, protocols and, of course, the compound itself. Even though one can imagine many ways to deliver compounds, it is reasonable to start to with a set of common ones that can be added to. Measurement requires the use of units; there are several kinds of units (time, mass, etc.) and several values for these units (hours, days, etc.), but these also can be easily recorded to build the ontology. Thus, one category of classes consists of those that can be built in a straightforward manner and without requiring extensive effort.

Another category of classes exists (e.g. compound); however, that cannot be enumerated without extensive effort; furthermore, many of these classes have been the focus of efforts by other groups to generate ontologies or various types of controlled vocabularies. For this second category of classes, the microarray community would be better served by having pointers to such resources rather than try to excerpt or recreate them. For example, unlike units or delivery methods, one cannot list the compounds that members of microarray community are likely to use because of the diversity of experiments that this community is interested in. Instead, the MGED ontology can refer to an external resource, such as ChemID*plus*,

Copyright © 2003 John Wiley & Sons, Ltd.

available from the National Library of Medicine, which includes 350 000 chemical records that can be searched by CAS Registry Number [4]. Another example of a class covered by an existing ontology is 'organism', for which the taxonomy available from the National Center for Biotechnology Information [22] can be used. A third illustrative example is 'disease'. For humans, multiple choices are available, such as GALEN [6], ICD-9 [9], SNOMED [18] and UMLS [21]. Having multiple choices raises the issue of how one chooses which is most appropriate to use and how one maps between the same values or terms in the different resources.

The approach taken in building the MGED ontology is to specify both types of classes, providing pointers to existing resources where appropriate, and providing explicit values for those classes that can be readily dealt with in this manner. Unlike other efforts, such as UMLS, that try to bring together different existing ontologies of the same domain (e.g. disease), the MGED ontology does not attempt to provide mappings between synonymous terms in different ontologies. This decision is based again on the limited resources of MGED and overwhelming diversity of domains for which such mappings would be required. Instead, the MGED ontology provides source information for these terms, allowing queries to identify when the terms came from different sources. These terms are provided with the class of OntologyEntry. For descriptions of the biological source (biosource) of the material used in the microarray experiment, a subclass BiosourceOntologyEntry was created. Properties of OntologyEntry include an association to a database entry that specifies the resource for terms for organism, disease, compound, etc.

The MGED ontology is being built with input from the microarray community and reflects the annotation needs of this community. The initial emphasis, therefore, has been on biomaterials used, and has included collecting the different annotation resources that investigators use for inclusion in OntologyEntry. The initial work has also included the manipulations that the biomaterial undergoes before collection (EnvironmentalHistory) and during treatment (Treatment). Also included are the manipulations for extracting RNA and generating labelled cDNAs (BiomaterialPreparation). The focus turned next to the experimental or study designs used and, most recently, an effort has begun to provide annotation for different kinds of microarrays (platform, substrate, etc.) and the protocols used to prepare and use them.

### Status and implementation of the ontology

The MGED ontology has been built using the OilEd tool [2] because it provides an expressive system covering both frame-based and description logics and it can import and export files into commonly used formats, such as RDFS and DAML + OIL. The current version of the ontology is freely available, with supporting documentation, from the MGED Ontology Working Group website [13]. Currently, changes are made to the MGED ontology only by the authors of this review; however, a discussion list [14] is used to propose changes and additions to the ontology.

MGED ontology version 1.6 contains 114 classes, 82 properties and 182 individuals. No axioms (constraints) have been included yet, as we want to flesh out the ontology as much as possible first. The ontology covers the complete description of the biomaterial used and the experimental or study design. Coverage of the microarray platform and protocols has begun and should be finished shortly. The target consumers of the MGED ontology are the investigators annotating experiments. Their primary interaction with the MGED ontology will be through web-based forms to enter annotation into a database. Such forms will use the MGED ontology to provide the fields that need entering, the dependencies between the fields, and the terms (in drop-down menus or associated tables) to populate those fields. It should be noted that the majority of people using the MGED ontology are different from the software and database developers using MAGE. The consequence is that development of the MGED ontology will be annotation-driven, whereas further development of MAGE is software-driven. Nonetheless, as MAGE does provide some semantics as well as syntactic standards, there is overlap with the MGED ontology and the two groups have joint discussions on such issues.

The authors of this review have begun using the MGED ontology in their own microarray gene expression database efforts (see Figure 1). These

Copyright © 2003 John Wiley & Sons, Ltd.

include a public repository for all microarray experiments (ArrayExpress, [1]) and integrated data systems that include microarray experiments for *Plasmodium falciparum* (PlasmoDB; [10, 16]) and the endocrine pancreas (EPConDB; [5]). PlasmoDB and EPConDB are supported by the GUS system [8], a relational database which now includes RAD [20]. RAD has been updated and modified to reflect the MGED standards. GUS also contains a shared resources component (SRes) that holds ontologies such as the MGED ontology. The RAD component contains a table called OntologyEntry that holds the components of the MGED ontology actually used (or needed) for the microarray experiments in RAD. The OntologyEntry table is used to populate project-specific forms with this information. For example, a PlasmoDB-specific form has been built that only includes those parts of the MGED ontology relevant to *Plasmodium*. The form is based on a generic template that is also customized for the EPConDB project. This approach seeks to streamline the annotation process by providing terms for relevant fields and eliminating irrelevant fields.

The ArrayExpress public repository for gene expression data [1] uses the MAGE model and aims to store MIAME-compliant data. In order to help users annotate and submit their data, a data submission tool, MIAMExpress [11], has been developed. MIAMExpress is an array platform — and experiment-type — independent tool that is based on the MIAME questionnaire. It consists of a series of web-based forms, and guides the user through data submission and annotation of the Experiment, the Protocols and the Array itself. The most challenging part of this annotation is the description of biological samples (Biomaterial) and their treatments (BiomaterialManipulation); therefore, instances from the MGED ontology have been used to populate the MIAMExpress forms which the user completes in order to annotate the experiment.

MIAMExpress is also a collection mechanism for terms that are used to annotate experiments. Users are offered the choice 'other' where no appropriate term exists, and are asked to provide a definition and a source for terms they provide. A source could be an existing external ontology (ExternalOntologyResource) or reference text. The ArrayExpress curators evaluate these terms and include them in MGED ontology where appropriate. This allows synonyms to be removed before inclusion in the MGED ontology and allows the ontology to grow in a user-driven manner. In future, versions of MIAMExpress species-specific interfaces will be developed, and terms from external ontologies will be included in addition to those from the MGED ontology, thus providing a unified interface for microarray annotation capable of using many ontologies. MIAMExpress is an open source project and is available for local installation as an annotation tool.

### Future of the ontology

The MGED ontology is now being developed to directly support MAGE-OM and will continue to grow as more microarray experiments are annotated and the terms needed for this purpose are shared. The MGED ontology will continue to develop into new areas, in keeping with the mission of the MGED society. Microarrays are used for purposes besides monitoring RNA abundance, and as the needs grow for annotating experiments generated by those other microarray applications, so will the impetus for including them in the MGED ontology. Microarray studies are only one of many approaches used in functional genomics that generate a large number of large datasets. Others, such as mass spectrometry-based proteomics, will also require standards and it is hoped that the experience gained and tools built for microarrays can be leveraged for other functional genomics approaches. In the case of the MGED ontology, the description of experimental samples and design should be generally applicable.

### Acknowledgements

The authors wish to thank Patricia Whetzel, Ele Holloway, Gaurab Mukherjee, Susanna Sansone and Philippe Rocca-Serra for critical reading of the manuscript, and contributions to the MGED ontology and the members of the Ontology Working Group for their input into this effort.

### References

1. ArrayExpress: **http://www.ebi.ac.uk.arrayexpress**
2. Bechhofer S, Horrocks I, Goble C, Stevens R. 2001. OilEd: a reason-able ontology editor for the semantic web. *Proceedings of KI2001* **2174**: 396–408.

Copyright © 2003 John Wiley & Sons, Ltd. 

3. Brazma A, Hingamp P, Quackenbush J, *et al*. 2001. Minimum information about a microarray experiment — MIAME — towards standards for microarray data. *Nature Genet* **29**: 365–371.
4. ChemID*plus*: **http://chem.sis.nlm.niv.gov/chemidplus/**
5. EPConDB: **http://www.cbil.upenn.edu/EPConDB**
6. GALEN: **http://www.opengalen.org/about.html**
7. Gruber TR. 1993. A translation approach to portable ontologies. *Knowledge Acquisition* **5**: 199–220.
8. GUS Platform: **http://www.gusdb.org**
9. ICD-9 (*International Classification of Diseases*, 9th edn): **http://www.cdc.gov/nchs/about/otheract/icd9/abticd9.htm**
10. Kissinger JC, Brunk BP, Crabtree J, *et al*. 2002. The *Plasmodium* genome database. *Nature* **419**: 490–492.
11. MIAMExpress: **http://www.ebi.ac.uk/miamexpress**
12. Microarray Gene Expression Data Society: **http://www.mged.org**
13. MGED Ontology Working Group: **http://www.mged.org/ontology**
14. MGED ontology discussion list: **http://lists.sourceforge.net/lists/listinfo/mged-ontologies**
15. Object Management Group: **http://www.omg.com/**
16. PlasmoDB: **http://plasmodb.org/**
17. Schena M, Shalon D, Davis RW, Brown PO. 1995. Quantitative monitoring of gene expression patterns with a complementary DNA microarray. *Science* **270**: 467–470.
18. SNOMED (Systematized Nomenclature of Medicine): **http://www.snomed.org/**
19. Spellman P, Miller M, Stewart J, *et al*. 2002. Design and implementation of microarray gene expression markup language (MAGE-ML). *Genome Biol* **3**: RESEARCH0046.
20. Stoeckert CJ, Pizarro A, Manduchi E, *et al*. 2001. A relational schema for array and non-array based gene expression data. *Bioinformatics* **17**: 300–308.
21. UMLS (Unified Medical Language System): **http://www.nlm.nih.gov/research/umls/**
22. Wheeler DL, Chappey C, Lash AE, *et al*. 2000. Database resources of the National Center for Biotechnology Information. *Nucleic Acids Res* **28**: 10–14.

Copyright © 2003 John Wiley & Sons, Ltd. 
